# Ultrasound-Guided Nerve Blocks for Flexor Tenosynovitis

**DOI:** 10.24908/pocus.v9i2.17395

**Published:** 2024-11-15

**Authors:** John M Bowling, Erick Zoumberakis

**Affiliations:** 1 Department of Emergency Medicine, Cleveland Akron General Akron, OH USA

**Keywords:** Ulnar, Intravenous Drug Use, Ultrasound-Guided Nerve Block, Point-of-Care Ultrasound, Emergency Department

## Abstract

Performing an ultrasound-guided nerve block (UGNB) is now common practice in many emergency departments (EDs) and is considered a core skill according to the American College of Emergency Physicians (ACEP). Nerve blocks are mostly utilized for fractures and laceration repairs, however, these blocks have many other applications. We present a case of utilizing an ulnar UGNB in a patient with flexor tenosynovitis and a history of intravenous drug use (IVDU) when parental opiates proved to be ineffective.

## Introduction

Performing an ultrasound-guided nerve block (UGNB) is now common practice in many emergency departments (EDs), especially academic programs with residents. Approximately 96 percent of surveyed academic emergency medicine programs perform UGNBs with the most common being the forearm nerve block (ulnar, median, and radial) 1. Performing these blocks is a core skill according to American College of Emergency Physicians (ACEP) 2. Fascial plane and peripheral UGNBs are most commonly utilized for fractures and laceration repairs, however, these blocks have many other applications. We present a case of utilizing an ulnar UGNB in a patient with flexor tenosynovitis and a history of intravenous drug use (IVDU) when parenteral opiates proved to be ineffective. At the time of our literature review, application of UGNBs for flexor tenosynovitis has never been reported.

## Case 

**Figure 1:**
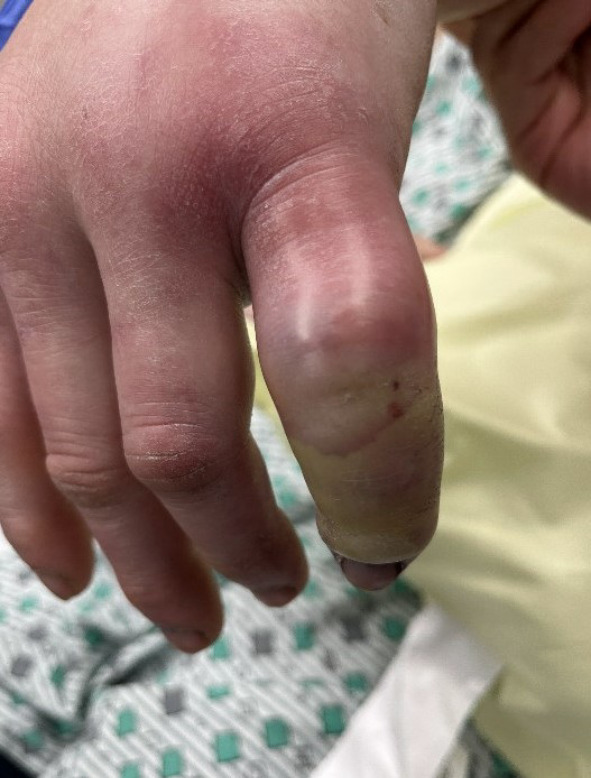
Left fifth digit of the patient.

**Figure 2:**
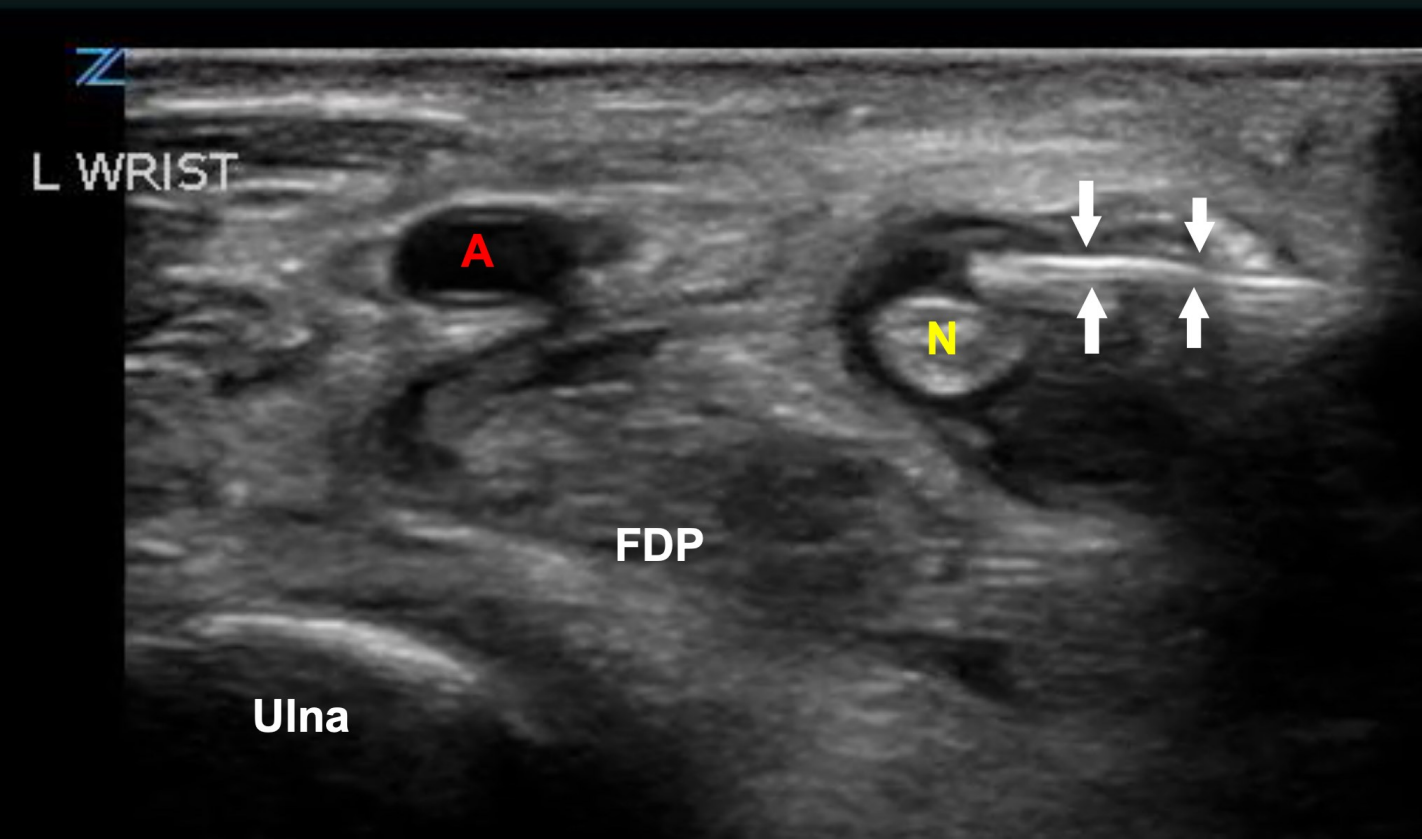
Point of care ultrasound image of the ulnar nerve block.

A 43-year-old male presented to the ED with severe left fifth digit pain after injuring his finger in a bicycle accident approximately two weeks prior. The patient endorsed a history of IVDU with fentanyl, most recently 12 hours before arrival. He denied any other injuries or injections into his digits. Upon clinical examination, the patient had all the Kanavel signs; his fifth digit was held in flexion, he had severe tenderness to the flexor tendon sheath, pain with motion, and a fusiform digit (Figure 1). X-ray was obtained and demonstrated a small fracture of the fifth middle phalanx. C-reactive protein and erythrocyte sedimentation rate were both elevated. The patient was started on ceftriaxone and vancomycin. He was subsequently admitted to the orthopedic service for operative management. While awaiting operative management in the ED, the patient had intractable pain despite aggressive treatment with multiple doses of parenteral hydromorphone. An ultrasound-guided nerve block, a non-opiate alternative pain management strategy, was discussed with the patient. Consent was obtained and an UGNB procedure was performed with Bupivacaine 0.5% (Figure 2 and Video S1). The patient received immediate relief and required minimal medication prior to operative management.

## Technique 

A: Ulnar Artery; N: Ulnar Nerve; White Arrows: Needle ; FPD: Flexor Digitorum Profundum Muscle 

**Figure 3:**
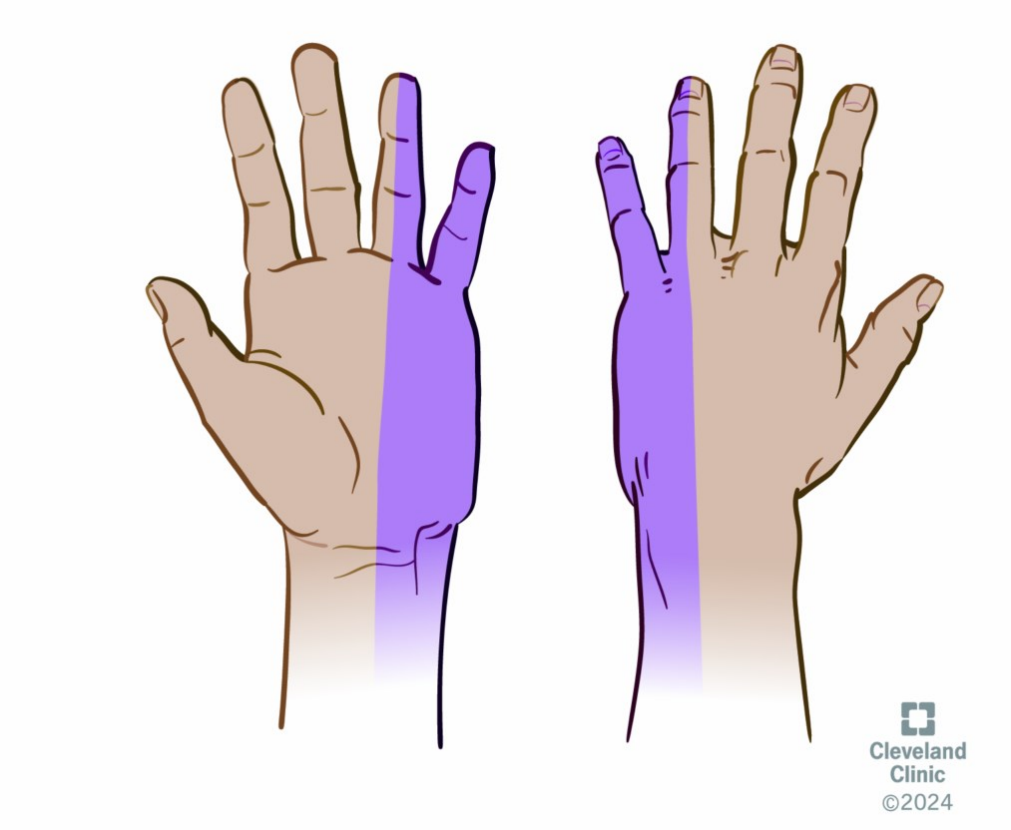
Distribution of the ulnar nerve block.

**Figure 4:**
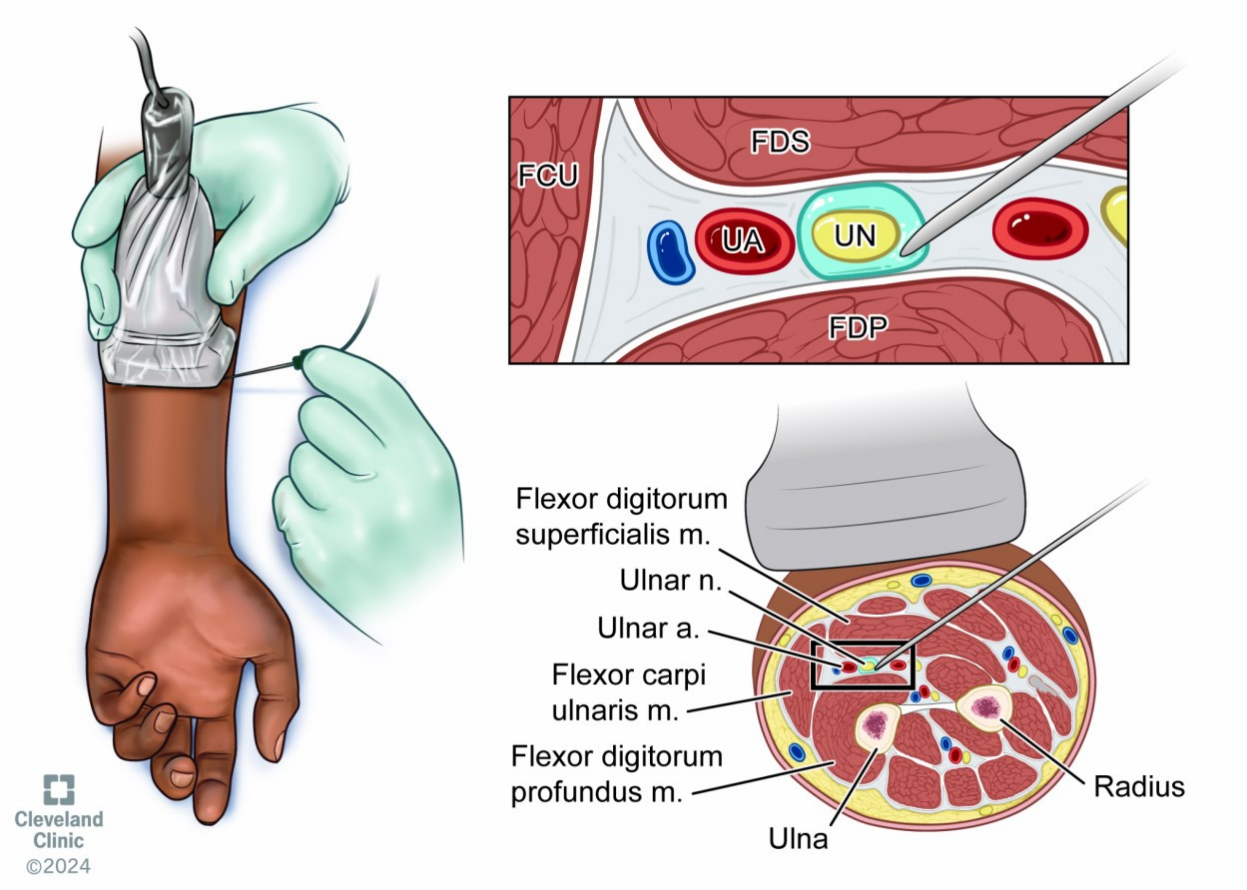
Anatomy and technique of the ulnar nerve block.

An ulnar UGNB was ideal for our patient as it anesthetized the ulnar aspect of the hand including the fifth digit and ulnar half of the fourth digit (Figure 3). This block requires 3 to 5 mL of bupivacaine 0.5%, or ropivacaine 0.5% along with a high frequency ultrasound probe, sterile gel and probe cover, 10 mL syringe, skin cleansing agent, and a 25 to 27 gauge needle. The steps to perform an ulnar UGNB are as follows:

1. Obtain consent and position the patient so that the medial aspect of the arm is easily accessible.

2. Utilize sterile technique and place the ultrasound probe with its sterile cover in the horizontal orientation of the medial aspect of the mid forearm, at least 5 cm from any area that involves the infection. The ulnar nerve should be easily identified as a honeycomb-like structure deep to the flexor carpi ulnaris (FCU) muscle and medial to the ulnar artery (Figure 4).

3. Insert the needle in-plane and use caution to avoid vascular structures. Confirm that the needle is not in a vascular space via aspiration.

4. Inject 1 to 2 mL at a time in the perineural space. Inject under the nerve first so if there is air in the needle, your view is not obscured. Avoid intraneural injection. Surround the whole nerve. If the patient endorses severe pain or if the needle tip is in the nerve, stop the injection and retract the needle.

Complications of this procedure can include infection, bleeding, hematoma, local anesthetic systemic toxicity (LAST), or nerve injuries including neuropraxia 3. However, visualizing the needle tip and utilizing ultrasound guidance helps to avoid complications 3.These common steps can be replicated for median and radial nerve blocks as well.

## Discussion

UGNBs provide a solution to challenging pain management scenarios that emergency medicine physicians often encounter. UGNBs have many applications such as in the case of flexor tenosynovitis. Flexor tenosynovitis is an exquisitely painful condition that occurs when purulent fluid collects between the visceral and parietal layers of the tendon, typically caused by trauma or hematologic spread. Classically, it is diagnosed clinically via the Kanavel signs 4, 5. Analgesic options can include non-opiates, opiates, warm compresses, and cryotherapy. In this case, our patient was desensitized to opiates given his history of IVDU, producing a challenging scenario.

## Conclusion

UGNBs are now an essential skill to emergency physicians and can greatly improve patient care. UGNBs are powerful tools that provide patients with the safest and most efficacious analgesia, especially in cases when parenteral opiates are ineffective. Our case demonstrates that these UGNBs have many other applications besides fractures and lacerations. 

## Patient Consent

Consent has been obtained from the patient for this case.

## Disclosure Statement 

The authors declare that they have no competing interests.
